# Fifteen years of irinotecan therapy for pediatric sarcoma: where to next?

**DOI:** 10.1186/s13569-015-0035-x

**Published:** 2015-08-28

**Authors:** Lars M. Wagner

**Affiliations:** Division of Pediatric Hematology/Oncology, Kentucky Clinic Suite, University of Kentucky, J-457, Lexington, KY 40536 USA

**Keywords:** Irinotecan, Sarcoma, Ewing sarcoma, Rhabdomyosarcoma

## Abstract

Over the past 15 years, irinotecan has emerged as an important agent for treating pediatric sarcoma patients. This review summarizes the activity noted in previous studies, and outlines current issues regarding scheduling, route of administration, and amelioration of side effects. Also discussed are new pegylated and nanoliposomal formulations of irinotecan and its active metabolite, SN-38, as well as future plans for how irinotecan may be used in combination with other conventional cytotoxic as well as targeted agents.

## Background

Irinotecan is a camptothecin analogue that has taken on growing importance in the treatment of pediatric sarcomas such as Ewing sarcoma and rhabdomyosarcoma. Irinotecan is a prodrug that is spontaneously converted by endogenous carboxylesterases to its active metabolite, SN-38. Like other camptothecins such as topotecan, SN-38 mediates cytotoxicity by stabilizing the DNA-topoisomerase I complex created during replication. This stabilization prevents religation of DNA, and so “poisons” the activity of the topoisomerase I enzyme.

Irinotecan was initially approved by the US Food and Drug Administration for the treatment of colon cancer in 1996. Three years later, Furman et al. reported the first pediatric phase I clinical trial of irinotecan [1]. This landmark study was based on the preclinical observation of improved efficacy when using a protracted multi-day schedule, as opposed to a single dose given every 3 weeks [2]. Such protracted scheduling provides greater exposure of this S phase-specific drug, especially when given for 5 consecutive days 2 weeks in a row (d × 5 × 2 schedule). The objective responses observed in three patients with relapsed rhabdomyosarcoma were consistent with the enhanced preclinical activity seen in pediatric sarcoma xenografts using this schedule, and this trial was followed by subsequent studies designed to: (1) explore various schedules of administration, (2) reduce toxicity, (3) improve convenience and maximize SN-38 exposures, and (4) define the activity of irinotecan as a single agent and in combination with other drugs. In this review, we will identify key findings from these past studies, and also discuss new formulations and potentially synergistic therapeutic partners for irinotecan.

## Schedules of irinotecan administration

Several schedules of irinotecan administration have been studied in children, ranging from one large dose every 3 weeks as used in adults [3, 4], to once weekly [5], daily × 3 [6], daily × 5 [7], and the original d × 5 × 2 schedule first studied by Furman et al. [1, 8, 9]. All schedules have been tolerable, although notably the pattern of toxicity is schedule-dependent. For example, when using larger but infrequent dosages, the principal toxicity is myelosuppression. In contrast, diarrhea and abdominal pain are more prominent with the protracted lower-dose schedule.

Only one pediatric study has directly compared the efficacy of different schedules of irinotecan. In that trial, 89 evaluable patients with recurrent rhabdomyosarcoma were randomized to receive vincristine combined with irinotecan given either on a d × 5 or a d × 5 × 2 schedule [10]. The overall incidence of grade 3–4 adverse events was similar. As expected, patients on the shorter schedule experienced more myelosuppression, while those on the longer schedule had more gastrointestinal toxicity. Importantly, because there was no significant difference in efficacy, and since the shorter schedule is more convenient and less expensive, the d × 5 schedule has emerged as the most popular schedule for newer regimens.

## Ameliorating toxicity

In most pediatric studies of irinotecan, myelosuppression is mild and growth factor is rarely required. Instead, diarrhea and abdominal pain are the usual dose-limiting toxicities. Early-onset diarrhea may occur during or immediately after irinotecan administration, and is usually manageable with atropine. More common and problematic is the late-onset diarrhea noted about 1 week after starting therapy. While prompt administration of loperamide may help with mild gastrointestinal toxicity, some patients experience severe diarrhea and abdominal pain, and this morbidity can impact compliance even when the tumor is responding to treatment [7, 8].

The mechanism of late-onset diarrhea is complex. Local accumulation of the active metabolite SN-38 in the gut results in direct cytotoxicity and secretory diarrhea [11]. SN-38 is usually inactivated through hepatic glucuronidation and then excreted in the bile into the intestine. However, reactivation of SN-38 can occur as a result of glucuronidases which are produced by enteric bacteria [reviewed in 12]. Therefore, one approach for reducing irinotecan-associated diarrhea is to use antibiotics to eradicate the Gram negative aerobic bacteria that produce these glucuronidases, thereby reducing the reactivation of local SN-38 in the gut. That strategy proved efficacious in a phase I trial of orally administered irinotecan in which the daily use of the oral cephalosporin cefixime reduced the incidence of grade 3–4 diarrhea such that the maximum tolerated dose was 50 % higher than what could be achieved without antibiotic support [13]. A 50 % increase in the tolerable dose was also noted in patients receiving intravenous irinotecan in a similar trial [14]. This practice of using cephalosporins before, during, and after the irinotecan course has now been universally employed in all pediatric trials of orally administered irinotecan, given that the poor bioavailability requires higher drug doses to achieve acceptable SN-38 exposures. One common approach when using the d × 5 schedule of irinotecan is to administer cephalosporins (either cefixime or cefpodoxime) starting 2 days before chemotherapy and continuing until 3 days after finishing chemotherapy, which makes for a 10-day course of antibiotics and avoids the continuous administration that may lead to antibiotic resistance or *C difficile* infections. In contrast to orally administered irinotecan, cephalosporin prophylaxis is not routinely done when standard doses of irinotecan are given intravenously, as the incidence of ≥grade 3 diarrhea is under 10 % [7]. Instead, antibiotic prophylaxis is usually only used in patients experiencing significant toxicity during the previous course, as a way to maintain dose intensity [15].

The detoxification of SN-38 through hepatic glucuronidaiton is mediated by *UGT1A1*. In adult studies, patients with the UGT1A1*28 polymorphism have increased toxicity from irinotecan [16]. However, in pediatric studies this genotype/phenotype relationship has not been observed. For example, in the largest series of 74 patients taken from 5 pediatric studies in patients receiving protracted irinotecan, there was no increase in either hematologic or gastrointestinal toxicity in patients homozygous for UGT1A1*28 [17]. Based on this and similar reports [18], prospective genotyping of pediatric patients receiving protracted irinotecan is not routinely performed.

## Maximizing convenience: oral administration

The protracted administration schedule of intravenous irinotecan is inconvenient for patients and costly to administer, prompting interest in oral administration. There is no commercially available tablet or capsule formulation of irinotecan, and so the intravenous preparation has been given orally. Because of the bitter taste, it is usually masked in cran-grape juice to improve palatability [13]. The oral bioavailability is less than 20 %, requiring higher dose of oral irinotecan are necessary to achieve SN-38 exposures similar to intravenous administration. However, metabolism of orally administered irinotecan is more efficient, given that the intestinal tract contains high levels of carboxylesterases, which may pre-systemically metabolize irinotecan to SN-38 and increase the SN-38/irinotecan ratio by threefold or more [19].

Pediatric clinical trials have shown the dose of 60 mg/m^2^/dose on a d × 5 × 2 schedule was tolerable and produced SN-38 exposures that were similar to those seen with intravenous doses of 20 mg/m^2^, when accounting for the wide intrapatient variability in irinotecan metabolism [13, 18]. However, the relationship between oral and intravenous dosing is not exactly linear. For example, the daily oral dose of 90 mg/m^2^ appears comparable to the intravenous dose of 50 mg/m^2^ when using similar pharmacokinetic assays [20]. To date there have been over 200 pediatric patients treated on trials of oral irinotecan [13, 18, 20–22]. Although there have been no studies directly comparing the efficacy of oral vs. intravenous administration, the roughly similar SN-38 exposures, response rates, and toxicity profiles suggest they are fairly equivalent when using the dose conversions noted above.

The benefits of oral administration include greater patient convenience and time away from the clinic, as well as up to five-fold reduction in cost [23]. The strategy is generally feasible, and because of the considerable benefits could be considered in most situations. However, there are occasional patients who have difficulty taking the medication orally, no matter what methods are used to mask the flavor. Also, for patients with ongoing nausea or chronic gastrointestinal complaints, oral absorption may be limited and make this strategy inappropriate.

## Improving SN-38 exposure

Efforts to increase SN-38 exposure are based on the assumption of a dose–response relationship for irinotecan therapy for pediatric sarcoma, which is intuitive but not yet proven clinically. Given gastrointestinal toxicity is the usual limiting toxicity, one strategy for dose escalation is to reduce irinotecan-associated diarrhea with cefixime as described above. McGreggor et al. have shown in a phase I trial this approach allows for an increase in intravenous irinotecan dosing from 20 to 30 mg/m^2^/day on the d × 5 × 2 schedule [14], although the efficacy of higher doses has not been formally assessed.

Another strategy to increase drug exposure is to reduce efflux of irinotecan out of cells by using the small molecule gefitinib to inhibit the ABCG2 drug transporter. Through this mechanism gefitinib can reverse irinotecan resistance in vitro even in cell lines that lack amplification of the epidermal growth factor receptor [24], which is the usual therapeutic target for this agent. ABCG2 is expressed in the small intestine, and co-administration of gefitinib can increase the bioavailability of oral irinotecan by four-fold [25]. Dose-finding studies of gefitinib in combination with both intravenous and oral irinotecan have been reported [22, 25], but there has not yet been efficacy assessment in a phase II trial.

## Activity of single-agent irinotecan

Single-agent irinotecan has been studied in a variety of pediatric trials. As predicted from mouse xenograft models [2, 26], responses have consistently been seen in patients with rhabdomyosarcoma and Ewing sarcoma. Response rates as high as 38 % for Ewing sarcoma/primitive neuroectodermal tumor and 16 % for rhabdomyosarcoma have been reported [9]. However, activity of single-agent irinotecan in larger multi-institutional phase II studies has been disappointing. For example, in a Children’s Oncology Group (COG) phase II trial using intravenous administration on a d × 5 schedule, response rates in relapsed patients were under 10 % for both rhabdomyosarcoma and Ewing sarcoma [7]. These results have led to the current practice of partnering irinotecan with another agent, such as vincristine or temozolomide, as described below. There is less experience using irinotecan for treatment of osteosarcoma or non-rhabdomyosarcoma soft tissue sarcoma, with only rare responses noted [20, 27].

## Identifying potential therapeutic partners

Preclinical experience shows camptothecins can synergize with microtubule inhibitors such as vincristine [28]. This combination has been most thoroughly evaluated in rhabdomyosarcoma, a disease in which vincristine is an established active agent. In newly-diagnosed patients with metastatic rhabdomyosarcoma, Pappo et al. reported a response rate of 42 % with single-agent irinotecan, which increased to 70 % when combined with vincristine [15]. The vincristine + irinotecan (VI) combination is tolerable, and a recent phase III trial for newly-diagnosed intermediate-risk rhabdomyosarcoma showed that incorporating cassettes of VI alternating with vincristine, dactinomycin, and cyclophosphamide (VAC) is as effective as using VAC alone, which had historically been the standard treatment for these patients [29]. As expected, febrile neutropenia and thrombocytopenia were less in patients receiving the VI cassettes, although there was more diarrhea. Moving forward, the COG is planning to use the VAC + VI regimen because it reduces the overall exposure to alkylating agents that may cause secondary malignancies and infertility.

Irinotecan has also been paired with the methylating agent temozolomide, given that modest myelosuppression seen from irinotecan allows for combination with drugs having more hematologic toxicity. Houghton et al. demonstrated schedule-dependent synergy with these two drugs against rhabdomyosarcoma xenografts [30], with maximum activity seen when temozolomide is given at least 1 h before irinotecan [31]. This is consistent with the proposed mechanism in which temozolomide-induced methylation of DNA causes localization of topoisomerase I-DNA complexes that are more susceptible to the cytotoxic effects of irinotecan [32]. This temozolomide + irinotecan (TI) combination has been particularly active in Ewing sarcoma, with reported response rates between 29 and 63 % [33–35]. The dose-limiting toxicities of irinotecan (diarrhea) and temozolomide (myelosuppression) are non-overlapping, and the combination is well-suited for oral administration. Because of the tolerability of this regimen, investigators have used TI as a backbone on which to add other drugs such as vincristine [20, 36, 37], as well as biologic agents discussed below.

A variety of other conventional chemotherapy agents have been combined with irinotecan to treat pediatric sarcoma, including carboplatin [38], oxaliplatin and/or gemcitabine [39–41], ifosfamide [42], and docetaxel [43]. None have achieved the response rates reported with VI or TI, and in some cases unexpected toxicities or pharmacokinetic interactions were seen. For example, although intermittent dosing of oxaliplatin and irinotecan was well tolerated in adults with colon cancer, severe pancreatic inflammation was seen when oxaliplatin was used together with protracted irinotecan in children [39]. Further, in a combination trial of ifosfamide and irinotecan for osteosarcoma patients, markedly reduced concentrations of SN-38 were noted, suggesting a major drug interaction that could compromise efficacy [42]. These findings demonstrate the importance of performing dose-finding and pharmacokinetic studies for novel combinations. A summary of published combination phase II and III studies of irinotecan-based regimens for pediatric sarcoma is provided in Table 1.

**Table 1:** Key phase II and III studies using irinotecan in pediatric sarcoma patients

Reference	Lead author	Phase	Other agents given with irinotecan	Population	Comments
[30]	Hawkins	III	Vincristine	Newly-diagnosed intermediate-risk RMS	VI alternating with VAC is as efficacious as VAC alone, and may reduce long-term toxicity
[15]	Pappo	II	Vincristine	Newly-diagnosed metastatic RMS	Response rate to induction rose from 46–70 % after addition of vincristine
[38]	Dharmajan	II	Carboplatin, radiation	Newly-diagnosed intermediate or high-risk RMS	Local control rate of 89 %; reduced mucositis compared to historical controls
[10]	Mascarenhas	II	Vincristine	Relapsed RMS	Similar rates of response and grade 3–4 toxicity between d × 5 vs d × 5 × 2 schedule
[37]	Mixon	II	Temozolomide, vincristine	Relapsed RMS	One complete response in 4 patients
[33]	Kurucu	II	Temozolomide	Relapsed ES	Response rate 55 %
[34]	Wagner	II	Temozolomide	Relapsed ES	Response rate 29 %
[35]	Casey	II	Temozolomide	Relapsed ES	Response rate 63 %
[36]	Raciborska	II	Temozolomide, vincristine	Relapsed ES	Response rate 68 %
[43]	Yoon	II	Docetaxel	Relapsed ES	Response rate 33 %
[42]	Crews	II	Ifosfamide	Newly-diagnosed high-risk osteosarcoma	Ifosfamide reduced SN-38 exposures

*RMS* rhabdomyosarcoma, *ES* Ewing sarcoma

## Future combinations to be explored

One focus in sarcoma therapeutics has been the addition of targeted agents onto conventional chemotherapy backbones. This strategy is particularly attractive if the targeted agent has either single-agent activity, or if it potentiates the cytotoxicity of standard chemotherapy drugs. An example is the addition of mTOR inhibitors such as temsirolimus to the TI regimen [21]. Responses in rhabdomyosarcoma patients to single-agent temsirolimus have been limited [44], but its combination with cyclophosphamide and vinorelbine showed promising activity in a recent COG trial [45]. Results from this study provided the rationale for the next upcoming COG phase III trial for intermediate-risk rhabdomyosarcoma, which will study the VAC/VI backbone with or without temsirolimus.

Another example is the combination of irinotecan-based regimens with a monoclonal antibody against the insulin growth factor receptor type I receptor (IGF-1R). Although the single-agent response rates to IGF-1R antibodies in phase II trials have been generally disappointing [reviewed in 46], there have been occasional patients with impressive and durable responses in patients with Ewing sarcoma and rhabdomyosarcoma [47, 48]. The COG has recently completed a phase II trial of the IGF-1R antibody cixutumumab together with multi-agent conventional chemotherapy for patients with newly-diagnosed metastatic rhabdomyosarcoma (ClinicalTrials.gov identifier NCT01055314). Interestingly, in the comparator arm of the study temozolomide was added on to the same chemotherapy backbone, which included irinotecan. Final results of this study are not yet available.

A third example is the use of inhibitors against the DNA repair protein poly(ADP-ribose) polymerase (PARP). This class of drugs was identified through a functional genomics approach and found to have marked preclinical in vitro and in vivo activity against Ewing sarcoma [49]. Although efficacy as monotherapy may be limited [50], the combination of a PARP inhibitor with temozolomide is now being explored in multiple trials, due to the potentiated effects of PARP inhibition following temozolomide-mediated DNA damage [51]. Stewart et al. have recently reported that further preclinical benefit may be seen by combining PARP inhibitors with both temozolomide and irinotecan [52].

Other molecular approaches include the targeting of Wee1, which helps regulate the response to DNA damage by inhibiting CDK1. Wee1 can be targeted with the small molecule MK-1775, which showed in vitro activity against a variety of sarcoma cell lines [53]. Combination with oral irinotecan is now being explored in a COG Phase I trial (ClinicalTrials.gov identifier NCT02095132), based on preclinical synergy with of this combination in neuroblastoma models [54].

It is important to note that not all irinotecan combinations may show benefit for sarcoma, even if used commonly for other tumor types. Although widely employed to treat high-grade glioma, the combination of irinotecan and the anti-VEGF antibody bevacizumab has shown no evidence to date of compelling activity in sarcoma in the limited studies to date [55, 56].

## New formulations of irinotecan and SN-38

The process of pegylation joins a drug with a multimeric polyethylene glycol using a glycine linker in order to prolong exposure to the agent. This approach has been applied in an effort to prolong the exposure to irinotecan and/or SN-38. These approaches are attractive in that preclinical studies have demonstrated responses even in irinotecan-resistant xenografts [57], and the schedule of administration is less frequent and therefore more convenient for patients. In a dose-finding study of pegylated SN-38 (EZN-2208), a maximum tolerated dose of 24 mg/m^2^ once every 3 weeks was identified, which was higher than the adult MTD of 16.5 mg/m^2^ [58]. Some gastrointestinal toxicity was seen at lower doses, with myelosuppression being dose-limiting at the higher doses. Unfortunately, no responses were seen in the 12 sarcoma patients treated on this phase I trial.

The pegylated irinotecan compound etirinotecan (NKTR-102) has shown promising activity in phase II studies of breast and ovarian cancer using a once every 3 weeks schedule [59, 60], and is moving forward in phase III trials. With this formulation, dehydration and diarrhea were the most common grade 3–4 toxicities, occurring in just over 20 % of patients. No trials have yet been reported which partner either of these drugs with other agents, and the long-term future of these agents likely awaits a review of their benefits in larger upcoming trials.

Liposomal preparations of irinotecan have also been developed, and may preferentially accumulate in tumor cells through enhanced permeability and retention [61]. Nanoliposomal irinotecan (MM-398) also minimizes exposure of drug in the serum and so stabilizes the active lactone form of irinotecan versus the inactive carboxylate form [62]. This drug has superior activity over comparably dosed conventional irinotecan in mouse models of Ewing sarcoma [63], and is currently being evaluated in a pediatric clinical trial together with cyclophosphamide (ClinicalTrials.gov identifier NCT02013336).

## Conclusions

The role of irinotecan in combination with other agents is becoming more established for the treatment of rhabdomyosarcoma, as well as for relapsed Ewing sarcoma. The d × 5 schedule may be as effective as more protracted administration, and is being used for many current and planned irinotecan trials. Oral administration is feasible for the majority of patients, may have similar activity and toxicity, and offers reduced cost and time away from the clinic. For these reasons, oral administration using a 5-day schedule is now commonly employed in the relapse setting at our institution, as well as in several ongoing clinical trials. Prophylaxis with cephalosporins is an important way to reduce severe irinotecan-associated diarrhea, and is necessary for all patients receiving oral administration of irinotecan. At present there is not a reliable way to identify patients at greatest risk of toxicity, and antibiotic prophylaxis is not routinely necessary for patients receiving intravenous irinotecan at standard doses. The single-agent activity of irinotecan is limited, although its toxicity profile allows for ready combination with a variety of other chemotherapy drugs, especially vincristine and temozolomide. Particularly exciting is the potential for combining irinotecan-based backbones with newer targeted therapies, and the opportunities for testing of the new longer-acting preparations either alone or in combination with other drugs.

## References

[1] Furman WL, Stewart CF, Poquette CA, Pratt CB, Santana VM, Zamboni WC (1999). Direct translation of a protracted irinotecan schedule from a xenograft model to a phase I trial in children. J Clin Oncol.

[2] Houghton PJ, Cheshire PJ, Hallman JD, Lutz L, Friedman HS, Danks MK (1995). Efficacy of topoisomerase I inhibitors, topotecan and irinotecan, administered at low dose levels in protracted schedules to mice bearing xenografts of human tumors. Cancer Chemother Pharmacol.

[3] Vassal G, Couanet D, Stockdale E, Geoffray A, Geoerger B, Orbach D (2007). Phase II trial of irinotecan in children with relapsed or refractory rhabdomyosarcoma: a joint study of the French Society of Pediatric Oncology and the United Kingdom Children’s Cancer Study Group. J Clin Oncol.

[4] Morland B, Platt K, Whelan JS (2014). A phase II window study of irinotecan (CPT-11) in high risk Ewing sarcoma: a Euro-E.W.I.N.G. study. Pediatr Blood Cancer.

[5] Bomgaars L, Kerr J, Berg S, Kuttesch J, Klenke R, Blaney SM (2006). A phase I study of irinotecan administered on a weekly schedule in pediatric patients. Pediatr Blood Cancer.

[6] Shitara T, Shimada A, Hanada R, Matsunaga T, Kawa K, Mugishima H (2006). Irinotecan for children with relapsed solid tumors. Pediatr Hematol Oncol.

[7] Bomgaars LR, Bernstein M, Krailo M, Kadota R, Das S, Chen Z (2007). Phase II trial of irinotecan in children with refractory solid tumors: a Children’s Oncology Group Study. J Clin Oncol.

[8] Cosetti M, Wexler LH, Calleja E, Trippett T, LaQuaglia M, Huvos AG (2002). Irinotecan for pediatric solid tumors: the Memorial Sloan-Kettering experience. J Pediatr Hematol Oncol.

[9] Bisogno G, Riccardi R, Ruggiero A, Arcamone G, Prete A, Surico G (2006). Phase II study of a protracted irinotecan schedule in children with refractory or recurrent soft tissue sarcoma. Cancer.

[10] Mascarenhas L, Lyden ER, Breitfeld PP, Walterhouse DO, Donaldson SS, Paidas CN (2010). Randomized phase II window trial of two schedules of irinotecan with vincristine in patients with first relapse or progression of rhabdomyosarcoma: a report from the Children’s Oncology Group. J Clin Oncol.

[11] Takasuna K, Hagiwara T, Hirohashi M, Kato M, Nomura M, Nagai E (1996). Involvement of beta-glucuronidase in intestinal microflora in the intestinal toxicity of the antitumor camptothecin derivative irinotecan hydrochloride (CPT-11) in rats. Cancer Res.

[12] Wagner LM, Crews KR, Stewart CF, Rodriguez-Galindo C, McNall-Knapp RY (2008). Reducing irinotecan-associated diarrhea in children. Pediatr Blood Cancer.

[13] Furman WL, Crews KR, Billups C, Wu J, Gajjar AJ, Daw NC (2006). Cefixime allows greater dose escalation of oral irinotecan: a phase I study in pediatric patients with refractory solid tumors. J Clin Oncol.

[14] McGregor LM, Stewart CF, Crews KR, Tagen M, Wozniak A, Wu J (2012). Dose escalation of intravenous irinotecan using oral cefpodoxime: a phase I study in pediatric patients with refractory solid tumors. Pediatr Blood Cancer.

[15] Pappo AS, Lyden E, Breitfeld P, Donaldson SS, Wiener E, Parham D (2007). Two consecutive phase II window trials of irinotecan alone or in combination with vincristine for the treatment of metastatic rhabdomyosarcoma: the Children’s Oncology Group. J Clin Oncol.

[16] O’Dwyer PJ, Catalano RB (2006). Uridine diphosphate glucuronosyltransferase (UGT) 1A1 and irinotecan: practical pharmacogenomics arrives in cancer therapy. J Clin Oncol.

[17] Stewart CF, Panetta JC, O’Shaughnessy MA, Throm SL, Fraga CH, Owens T (2007). UGT1A1 promoter genotype correlates with SN-38 pharmacokinetics, but not severe toxicity in patients receiving low-dose irinotecan. J Clin Oncol.

[18] Wagner LM, Villablanca JG, Stewart CF, Crews KR, Groshen S, Reynolds CP (2009). Phase I trial of oral irinotecan and temozolomide for children with relapsed high-risk neuroblastoma: a new approach to neuroblastoma therapy consortium study. J Clin Oncol.

[19] Drengler RL, Kuhn JG, Schaaf LJ, Rodriguez GI, Villalona-Calero MA, Hammond LA (1999). Phase I and pharmacokinetic trial of oral irinotecan administered daily for 5 days every 3 weeks in patients with solid tumors. J Clin Oncol.

[20] Wagner LM, Perentesis JP, Reid JM, Ames MM, Safgren SL, Nelson MD (2010). Phase I trial of two schedules of vincristine, oral irinotecan, and temozolomide (VOIT) for children with relapsed or refractory solid tumors: a Children’s Oncology Group phase I consortium study. Pediatr Blood Cancer.

[21] Bagatell R, Norris R, Ingle AM, Ahern C, Voss S, Fox E (2014). Phase 1 trial of temsirolimus in combination with irinotecan and temozolomide in children, adolescents and young adults with relapsed or refractory solid tumors: a Children’s Oncology Group Study. Pediatr Blood Cancer.

[22] Brennan RC, Furman W, Mao S, Wu J, Turner DC, Stewart CF (2014). Phase I dose escalation and pharmacokinetic study of oral gefitinib and irinotecan in children with refractory solid tumors. Cancer Chemother Pharmacol.

[23] Wagner LM (2010). Oral irinotecan for treatment of pediatric solid tumors: ready for prime time?. Pediatr Blood Cancer.

[24] Stewart CF, Leggas M, Schuetz JD, Panetta JC, Cheshire PJ, Peterson J (2004). Gefitinib enhances the antitumor activity and oral bioavailability of irinotecan in mice. Cancer Res.

[25] Furman WL, Navid F, Daw NC, McCarville MB, McGregor LM, Spunt SL (2009). Tyrosine kinase inhibitor enhances the bioavailability of oral irinotecan in pediatric patients with refractory solid tumors. J Clin Oncol.

[26] Vassal G, Terrier-Lacombe MJ, Bissery MC, Vénuat AM, Gyergyay F, Bénard J (1996). Therapeutic activity of CPT-11, a DNA-topoisomerase I inhibitor, against peripheral primitive neuroectodermal tumour and neuroblastoma xenografts. Br J Cancer.

[27] McNall-Knapp RY, Williams CN, Reeves EN, Heideman RL, Meyer WH (2010). Extended phase I evaluation of vincristine, irinotecan, temozolomide, and antibiotic in children with refractory solid tumors. Pediatr Blood Cancer.

[28] Thompson J, George EO, Poquette CA, Cheshire PJ, Richmond LB, de Graaf SS (1999). Synergy of topotecan in combination with vincristine for treatment of pediatric solid tumor xenografts. Clin Cancer Res.

[29] Hawkins DS, Anderson JR, Mascarenhas L, McGowage GB, Rodeberg DA, Wolden SL (2014). Vincristine, dactinomycin, cyclophosphamide (VAC versus VAC/V plus irinotecan for intermediate-risk rhabdomyosarcoma: a report from the Children’s Oncology Group Soft Tissue sarcoma Committee. J Clin Oncol.

[30] Houghton PJ, Stewart CF, Cheshire PJ, Richmond LB, Kirstein MN, Poquette CA (2000). Antitumor activity of temozolomide combined with irinotecan is partly independent of O6-methylguanine-DNA methyltransferase and mismatch repair phenotypes in xenograft models. Clin Cancer Res.

[31] Patel VJ, Elion GB, Houghton PJ, Keir S, Pegg AE, Johnson SP (2000). Schedule-dependent activity of temozolomide plus CPT-11 against a human central nervous system tumor-derived xenograft. Clin Cancer Res.

[32] Pourquier P, Waltman JL, Urasaki Y, Loktionova NA, Pegg AE, Nitiss JL (2001). Topoisomerase I-mediated cytotoxicity of *N*-methyl-*N*′-nitro-*N*-nitrosoguanidine: trapping of topoisomerase I by the O6-methylguanine. Cancer Res.

[33] Kurucu N, Sari N, Ilhan IE (2015). Irinotecan and temozolamide treatment for relapsed Ewing sarcoma: a single-center experience and review of the literature. Pediatr Hematol Oncol.

[34] Wagner LM, McAllister N, Goldsby RE, Rausen AR, McNall-Knapp RY, McCarville MB (2007). Temozolomide and intravenous irinotecan for treatment of advanced Ewing sarcoma. Pediatr Blood Cancer.

[35] Casey DA, Wexler LH, Merchant MS, Chou AJ, Merola PR, Price AP (2009). Irinotecan and temozolomide for Ewing sarcoma: the Memorial Sloan-Kettering experience. Pediatr Blood Cancer.

[36] Raciborska A, Bilska K, Drabko K, Chaber R, Pogorzala M, Wyrobek E (2013). Vincristine, irinotecan, and temozolomide in patients with relapsed and refractory Ewing sarcoma. Pediatr Blood Cancer.

[37] Mixon BA, Eckrich MJ, Lowas S, Engel ME (2013). Vincristine, irinotecan, and temozolomide for treatment of relapsed alveolar rhabdomyosarcoma. J Pediatr Hematol Oncol.

[38] Dharmarajan KV, Wexler LH, Wolden SL (2013). Concurrent radiation with irinotecan and carboplatin in intermediate- and high-risk rhabdomyosarcoma: a report on toxicity and efficacy from a prospective pilot phase II study. Pediatr Blood Cancer.

[39] McGregor LM, Spunt SL, Furman WL, Stewart CF, Schaiquevich P, Krailo MD (2009). Phase 1 study of oxaliplatin and irinotecan in pediatric patients with refractory solid tumors: a children’s oncology group study. Cancer.

[40] Hartmann C, Weinel P, Schmid H, Grigull L, Sander A, Linderkamp C (2011). Oxaliplatin, irinotecan, and gemcitabine: a novel combination in the therapy of progressed, relapsed, or refractory tumors in children. J Pediatr Hematol Oncol.

[41] Zak D, Styler MJ, Rosenbluth JZ, Brodsky I (2005). Combination of gemcitabine and irinotecan for recurrent metastatic osteogenic sarcoma. Clin Adv Hematol Oncol.

[42] Crews KR, Stewart CF, Liu T, Rodriguez-Galindo C, Santana VM, Daw NC (2004). Effect of fractionated ifosfamide on the pharmacokinetics of irinotecan in pediatric patients with osteosarcoma. J Pediatr Hematol Oncol.

[43] Yoon JH, Kwon MM, Park HJ, Park SY, Lim KY, Joo J (2014). A study of docetaxel and irinotecan in children and young adults with recurrent or refractory Ewing sarcoma family of tumors. BMC Cancer.

[44] Geoerger B, Kieran MW, Grupp S, Perek D, Clancy J, Krygowski M (2012). Phase II trial of temsirolimus in children with high-grade glioma, neuroblastoma, and rhabdomyosarcoma. Eur J Cancer.

[45] Mascarenhas L, Meyer WH, Lyden E, Rodeberg DA, Indelicato DJ, Linardic CM (2014). Randomized phase II trial of bevacizumab and temsirolimus in combination with vinorelbine and cyclophosphamide for first relapse/disease progression of rhabdomyosarcoma: a report from the Children’s Oncology Group. J Clin Oncol.

[46] Olmos D, Tan DS, Jones RL, Judson IR (2010). Biological rationale and current clinical experience with anti-insulin-like growth factor 1 receptor monoclonal antibodies in treating sarcoma: twenty years from the bench to the bedside. Cancer J.

[47] Malempati S, Weigel B, Ingle AM, Ahern CH, Carroll JM, Roberts CT (2012). Phase I/II trial and pharmacokinetic study of cixutumumab in pediatric patients with refractory solid tumors and Ewing sarcoma: a report from the Children’s Oncology Group. J Clin Oncol.

[48] Weigel B, Malempati S, Reid JM, Voss SD, Cho SY, Chen HX (2014). Phase 2 trial of cixutumumab in children, adolescents, and young adults with refractory solid tumors: a report from the Children’s Oncology Group. Pediatr Blood Cancer.

[49] Garnett MJ, Edelman EJ, Heidorn SJ, Greenman CD, Dastur A, Lau KW (2012). Systematic identification of genomic markers of drug sensitivity in cancer cells. Nature.

[50] Choy E, Butrynski JE, Harmon DC, Morgan JA, George S, Wagner AJ (2014). Phase II study of olaparib in patients with refractory Ewing sarcoma following failure of standard chemotherapy. BMC Cancer.

[51] Smith MA, Reynolds CP, Kang MH, Kolb EA, Gorlick R, Carol H (2015). Synergistic activity of PARP inhibition by talazoparib (BMN 673) with temozolomide in pediatric cancer models in the pediatric preclinical testing program. Clin Cancer Res.

[52] Stewart E, Goshorn R, Bradley C, Griffiths LM, Benavente C, Twarog NR (2014). Targeting the DNA repair pathway in Ewing sarcoma. Cell Rep.

[53] Kreahling JM, Gemmer JY, Reed D, Letson D, Bui M, Altiok S (2012). MK1775, a selective Wee1 inhibitor, shows single-agent antitumor activity against sarcoma cells. Mol Cancer Ther.

[54] Russell MR, Levin K, Rader J, Belcastro L, Li Y, Martinez D (2013). Combination therapy targeting the Chk1 and Wee1 kinases shows therapeutic efficacy in neuroblastoma. Cancer Res.

[55] Wagner L, Turpin B, Nagarajan R, Weiss B, Cripe T, Geller J (2013). Pilot study of vincristine, oral irinotecan, and temozolomide (VOIT regimen) combined with bevacizumab in pediatric patients with recurrent solid tumors or brain tumors. Pediatr Blood Cancer.

[56] Okada K, Yamasaki K, Tanaka C, Fujisaki H, Osugi Y, Hara J (2013). Phase I study of bevacizumab plus irinotecan in pediatric patients with recurrent/refractory solid tumors. Jpn J Clin Oncol.

[57] Pastorino F, Loi M, Sapra P, Becherini P, Cilli M, Emionite L (2010). Tumor regression and curability of preclinical neuroblastoma models by PEGylated SN38 (EZN-2208), a novel topoisomerase I inhibitor. Clin Cancer Res.

[58] Norris RE, Shusterman S, Gore L, Muscal JA, Macy ME, Fox E (2014). Phase 1 evaluation of EZN-2208, a polyethylene glycol conjugate of SN38, in children adolescents and young adults with relapsed or refractory solid tumors. Pediatr Blood Cancer.

[59] Awada A, Garcia AA, Chan S, Jerusalem GH, Coleman RE, Huizing MT (2013). Two schedules of etirinotecan pegol (NKTR-102) in patients with previously treated metastatic breast cancer: a randomised phase 2 study. Lancet Oncol.

[60] Vergote IB, Garcia A, Micha J, Pippitt C, Bendell J, Spitz D (2013). Randomized multicenter phase II trial comparing two schedules of etirinotecan pegol (NKTR-102) in women with recurrent platinum-resistant/refractory epithelial ovarian cancer. J Clin Oncol.

[61] Kalra AV, Kim J, Klinz SG, Paz N, Cain J, Drummond DC, Nielsen UB, Fitzgerald JB (2014). Preclinical activity of nanoliposomal irinotecan is governed by tumor deposition and intratumor prodrug conversion. Cancer Res.

[62] Drummond DC, Noble CO, Guo Z, Hong K, Park JW, Kirpotin DB (2006). Development of a highly active nanoliposomal irinotecan using a novel intraliposomal stabilization strategy. Cancer Res.

[63] Kang MH, Wang J, Makena MR, Lee JS, Paz N, Hall CP (2015). Activity of MM-398, nanoliposomal irinotecan (nal-IRI), in Ewing’s family tumor xenografts is associated with high exposure of tumor to drug and high SLFN11 expression. Clin Cancer Res.

